# Reply to Grant, W.B. Comment on “Coelho-Junior et al. Protein Intake and Frailty in Older Adults: A Systematic Review and Meta-Analysis of Observational Studies. *Nutrients* 2022, *14*, 2767”

**DOI:** 10.3390/nu14224881

**Published:** 2022-11-18

**Authors:** Hélio José Coelho-Junior, Riccardo Calvani, Anna Picca, Matteo Tosato, Francesco Landi, Emanuele Marzetti

**Affiliations:** 1 Department of Geriatrics and Orthopedics, Università Cattolica del Sacro Cuore, 00168 Rome, Italy; 2 Fondazione Policlinico Universitario “Agostino Gemelli” IRCCS, L.go A. Gemelli 8, 00168 Rome, Italy; 3 Department of Medicine and Surgery, LUM University, 70100 Casamassima, Italy

We recently conducted a systematic review and meta-analysis to assess the association between protein consumption and frailty in older adults [1]. Findings indicated that protein consumption was longitudinally, but not cross-sectionally, associated with frailty status. In addition, a stratified analysis according to protein sources was conducted based on three cross-sectional studies that provided these data. Results showed that frail people consumed less animal protein, but no plant-based protein, than robust older adults. Grant [2] mentioned that our discussion was based on animal protein digestibility and branched-chain amino acid content (BCAA), whereas the impact of vitamin D (vitD) on frailty was omitted. We believe that some important topics should be further discussed. 

We respectfully disagree with Grant [2] that vitD is the main mediator in the relationship between animal products and frailty. As we mentioned in our article [1], the intake of animal-based protein is highly encouraged in older adults owing to their metabolic and biochemical properties [3]. Specifically, animal-derived protein is rich in essential amino acids, including BCAA [3,4]. Smith et al. [5] observed that the administration of non-essential amino acids did not evoke muscle protein synthesis, whereas muscle anabolism rates near 90% were observed after the infusion of essential amino acids. Among essential amino acids, much attention has been paid to BCAAs and mainly leucine, which is recognized as a major stimulator of muscle anabolism. At the molecular level, leucine increases muscle anabolism by activating the mammalian target of rapamycin [6] and its downstream proteins [6,7]. 

The association between leucine and frailty-related parameters has been explored in several studies. Lixandrão et al. [8] found a significant relationship between dietary leucine intake and lower-limb muscle strength and mass in Brazilian older adults. Coelho-Junior et al. [9] found that leucine intake was significantly associated with muscle strength and power in a sample of Italian older adults. After examining a large cohort of Korean older adults, Park et al. [10] detected a positive association between leucine intake and handgrip strength. McDonald et al. [11] reported that older Danish adults with greater leucine intake lost less lean body mass during a six-year follow-up. 

An interesting point of view was provided by Pikosky et al. [12]. The authors observed that handgrip strength increased according to the intake of dietary animal and vegetal protein and leucine. However, stronger older adults consumed more animal protein and leucine. A recent systematic review of randomized controlled trials that examined approximately 700 older adults indicated that leucine-rich protein supplements significantly increased muscle strength [13]. 

An increasing number of studies investigated the relationship between frailty and BCAA ingestion. Coelho-Junior et al. [14] observed that robust and prefrail older adults consumed more BCAAs than their robust counterparts. A recent study noted that physical exercise plus BCAA supplementation might reduce physical performance loss during the detraining period in frail older adults [15]. Such results suggest that animal protein and its biochemical properties have an important role in counteracting frailty. 

The association between vitD and frailty also deserves discussion. A recent systematic review and meta-analysis found that circulating vitD was not a biomarker shared between sarcopenia and frailty, which suggests that vitD may not be involved in the pathogenesis of physical frailty [16]. Furthermore, several large randomized clinical trials found no effects of vitD supplementation on frailty-related parameters. For instance, Bischoff-Ferrari et al. [17] observed that a three-year treatment with vitD did not ameliorate short physical performance battery (SPPB) scores, cognition, or the incidences of nonvertebral fractures in septuagenarians. Uusi-Rasi et al. [18] reported that supplementation with vitD did not reduce the incidence of falls and injurious falls in older women. The effects of vitD, combined with exercise training, were also tested. Mølmen et al. [19] observed that vitD supplementation did not increase the effects of resistance training on muscle mass and strength. An interesting critical review of large randomized clinical trials (n > 2000 participants in each) was offered by Scragg and Sluyter [20], who concluded that the effects of vitD supplementation on muscle health are minimal or null. 

Results of meta-analyses have confirmed the limited effects of vitD supplementation on frailty-related parameters. Beaudart et al. [21] showed that vitD supplementation had small but significant effects on global muscle strength. However, no significant changes were observed in muscle mass or power. Similarly, Muir and Montero-Odasso [22] reported that vitD might improve muscle strength, but not mobility, in older adults. An elegant trial-level meta-analysis of placebo-controlled trials was conducted by Bislev et al. [23]. The authors observed that, when compared to placebo, supplementation with vitD was associated with a trend toward worsening performance on the SPPB, timed “Up and Go!”, and muscle strength tests, whereas no effects were observed on muscle mass or mobility. 

An important point raised by several authors is that the effects of vitD supplementation might be more pronounced in people with vitD deficiency [20,23]. However, an individual participant meta-analysis [24] found that vitD supplementation did not affect muscle strength and power, mobility, balance, or body composition in those with vitD insufficiency. 

Collectively, available data indicate that the positive association between vitD and frailty-related parameters may be limited to observational studies, which do not prove causative effects. The lack of experimental studies and paired and network meta-analyses does not allow us either confuting or supporting Grant’s assumptions on the superiority of vitD relative to dietary protein.

Another important aspect to consider is that sunlight exposure is the major source of vitD [25,26]. In contrast, very few foods are naturally rich in the inactive and active forms of vitD, and some distinguished examples include fish food and cod liver oil [25,26]. The amount of vitD produced in response to five to 10 min of sunlight exposure in adequate environments is equivalent to ingesting approximately 300 g of fresh salmon [25,26]. Such evidence led experts in the field to propose that dietary vitD is unnecessary if people adequately expose themselves to sunlight [27]. 

Dietary sources of vitD are also very expensive. In addition, meat consumption is a complicated issue in older adults, mainly in those with frailty, due to socioeconomic and cultural aspects [28] and oral health problems [29]. In fact, dairy and processed meat products are the main sources of animal protein consumed by older adults [30]. Hence, it is plausible that the relationship between animal protein and frailty is more dependent on BCAA content and availability than on vitD intake through the diet.

Our meta-analytic results were not stratified according to vitD levels because most studies did not report these data. An interesting perspective for future studies would be to use dietary vitD, and mainly sunlight exposure, as a moderator in the association between animal protein intake and frailty. The prevalence of participants supplemented with vitD should also be taken into consideration.

Finally, adequate consumption of plant protein might be associated with frailty-related parameters in older adults [31,32]. In addition, studies are urgently required to confirm if sunlight exposure properly stimulates vitD production in vegetarian and vegan people to achieve optimal endogenous levels of vitD. If so, adequate sunlight exposure, and not vitD supplementation, should be advised. Taking a sunbath is definitely more fun than ingesting a pill, after all!
